# Treatment Options for Advanced Non-Small Cell Lung Cancer After Failure of Previous Immune Checkpoint Inhibitors and Chemotherapy: Meta-Analysis of Five Randomized Controlled Trials

**DOI:** 10.3390/curroncol32010046

**Published:** 2025-01-17

**Authors:** Andrea Messori, Andrea Ossato, Lorenzo Gasperoni, Luna Del Bono, Alessandro Inno, Vera Damuzzo

**Affiliations:** 1 HTA Unit, Regional Health Service, 50139 Florence, Italy; 2 Department of Pharmaceutical and Pharmacological Sciences, University of Padua, 35131 Padova, Italy; 3 Oncological Pharmacy Unit, IRCCS Istituto Romagnolo per lo Studio dei Tumori (IRST) “Dino Amadori”, 47014 Meldola, Italy; lorenzo.gasperoni@irst.emr.it; 4 School of Specialization in Hospital Pharmacy, Department of Pharmacy, University of Pisa, 56126 Pisa, Italy; l.delbono3@studenti.unipi.it; 5 Medical Oncology, IRCCS Ospedale Sacro Cuore Don Calabria, 37024 Negrar di Valpolicella, Italy; 6 Hospital Pharmacy, Vittorio Veneto Hospital, 31029 Vittorio Veneto, Italy

**Keywords:** immune checkpoint inhibitors, biomarkers, resistance mechanisms, non-small cell lung cancer

## Abstract

Background: Immune checkpoint inhibitors (ICIs), either alone or in combination with platinum-based chemotherapy, are effective in the first-line treatment of metastatic, non-oncogene-addicted, non-small cell lung cancer (NSCLC). However, when NSCLC patients progress, the efficacy of available treatment options is limited. Methods: We undertook a meta-analysis that compared combination regimens with the current standard of care. Only randomized controlled trials (RCTs) were included (endpoint, overall survival [OS]). Our analysis used an artificial intelligence software program that reconstructs individual patient data from Kaplan–Meier curves. Hazard ratio (HR) with 95% confidence interval (CI) was the main parameter. Heterogeneity was based on Wald’s test and likelihood ratio test. Results: Five RCTs were included, whose experimental arms included five different combinations. In our analysis, these combination regimes showed no OS benefit compared to chemotherapy (HR = 1.066, 95%CI, 0.9311 to 1.221; *p* = 0.35). Among the five control arms, cross-trial heterogeneity was remarkably low (likelihood ratio test = 3.76 on 4 df, *p* = 0.40; Wald test = 3.83 on 4 df, *p* = 0.40. Discussion: In conclusion, five new second-line combination treatments for patients with NSCLC were not found to determine any benefit in terms of OS in comparison with the current standard of care.

## 1. Introduction

Standard first-line treatment for patients with stage IV non-small cell lung cancer (NSCLC) without druggable molecular alterations is represented by immune checkpoint inhibitors (ICIs) alone—if programmed death ligand-1 (PD-L1) expression level is ≥50%—or—for any PD-L1 expression level—by chemo–immunotherapy combinations consisting of ICIs plus platinum-based chemotherapy [1]. Regarding chemo–immunotherapy, several combinations of platinum-based histology-driven chemotherapy regimens with different ICIs are available, including pembrolizumab [2,3], atezolizumab with or without bevacizumab [4,5], nivolumab and ipilimumab [6], cemiplimab [7], and durvalumab plus tremelimumab [8].

Although ICIs alone or in combination with platinum-based chemotherapy have demonstrated survival benefits as first-line treatment in patients with metastatic, non-oncogene-addicted NSCLC, the disease may still ultimately progress [1]. Before the advent of ICIs in the front-line setting, docetaxel (either alone or in combination with antiangiogenic drugs like nintedanib for adenocarcinoma histology or ramucirumab for all histologies) was considered the standard of care after disease progression on platinum-based chemotherapy. Other single-agent chemotherapies such as paclitaxel, gemcitabine, vinorelbine, or pemetrexed (if not already included in first-line combinations) have shown comparable efficacy [9], and are often used in clinical practice as an alternative to docetaxel as second-line treatment or beyond. Moreover, data on single-agent chemotherapy primarily refer to patients pre-treated with platinum-based chemotherapy, while data for patients pre-treated with ICIs are extremely limited. Re-challenge with ICIs has also been explored, but data are still inconclusive [10,11]; thus, single-agent chemotherapy for patients pretreated with both ICIs and platinum-based chemotherapy remains the standard. Therefore, clinical research is actively evaluating innovative combinations compared to standard chemotherapy for patients whose disease progresses after ICIs +/− platinum-based chemotherapy.

In this study, we evaluated all randomized controlled trials (RCTs) that evaluated new combination treatments as second-line or beyond for patients with disease progression after ICIs and platinum-based chemotherapy. Our analysis was aimed at assessing whether these pooled combination treatments can confer a survival benefit in terms of overall survival (OS) compared with the standard of care. In addition, to overcome the lack of direct comparison between the many potential new pharmacological treatment combinations, we aimed to assess whether any of the combination treatments can confer a statistically significant survival benefit in terms of OS compared to standard of care.

Wang et al. have recently published a network meta-analysis on this topic in which all assessments of OS [12], typically reported in a Forest plot, were binary and, therefore, did not consider the timing at which events occurred and, accordingly, did not consider the follow-up duration of individual trials.

In the present study, we used a relatively new evidence-based method (the IPDfromKM method [13]) to provide specific statistical support for the above conclusion given that the presence of a survival benefit with combination therapies is a matter of controversy. Published in June 2021 by Liu et al., the IPDfromKM method uses automated techniques to derive individual patient data from the Kaplan–Meier survival curves resulting from RCTs. This reconstructed data allow for indirect comparisons of treatments across studies through standard statistical approaches, like the Cox regression. In addition, the methods allow patients treated with the same regimen but enrolled in different RCTs to be pooled, thereby increasing the sample size, accounting for differences in the length of follow-up, and ultimately providing a more comprehensive assessment of time-to-event outcomes. The survival curve of each regimen included in the analysis is plotted on a multi-treatment Kaplan–Meier plot, which provides an immediate summary of the results.

This method has already found numerous successful applications, particularly in the fields of oncology [14] and cardiology [15].

## 2. Materials and Methods

Literature search. We searched PubMed, Scopus, and EMBASE to identify randomized controlled trials (RCTs) that met the eligibility criteria for the analysis. The final search was conducted on the 10th of August 2024. The search term was constructed as follows: [“non-small cell lung cancer” OR “NSCLC” OR “non-small-cell lung cancer”) AND (“advanced” OR “locally advanced” OR “metastatic”) AND (“failure” OR “progression” OR “relapse” OR “after” OR “second-line therapy” OR “subsequent therapy” + filter “published in the last 5 years”]. The selection of articles was conducted in accordance with the PRISMA algorithm [16].

**Figure 1:**
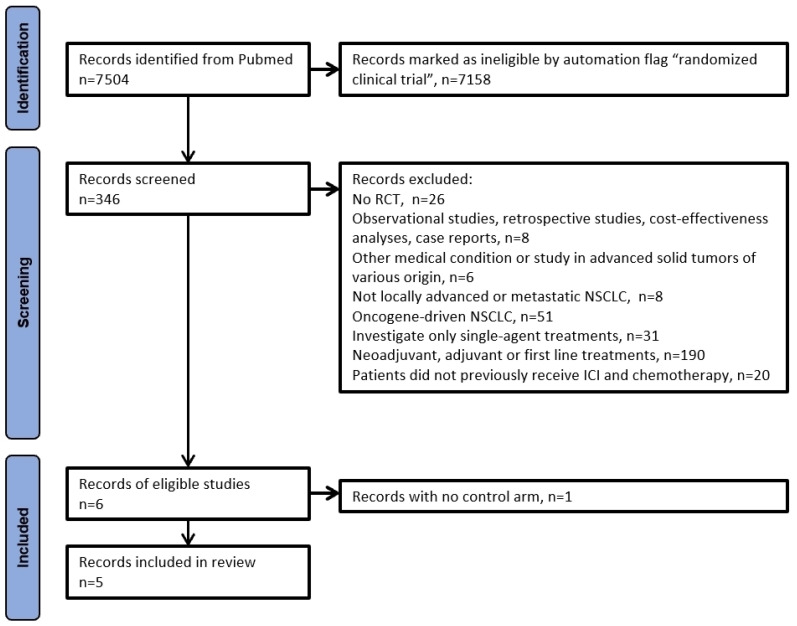
PRISMA flowchart of our literature search. The keywords used for our initial search were as follows: Pubmed “non-small cell lung cancer” OR “NSCLC” OR “non-small-cell lung cancer”) AND (“advanced” OR “locally advanced” OR “metastatic”) AND (“failure” OR “progression” OR “relapse” OR “after” OR “second-line therapy” OR “subsequent therapy” + filter “published in the last 5 years”.

Our inclusion criteria were represented by patients with non-oncogenic driven, unresectable locally advanced, or metastatic NSCLC who experienced disease progression after immune checkpoint inhibitors and chemotherapy. The primary endpoint of our analysis was OS, secondary endpoint was PFS. We included all RCTs that tested a combination treatment in the experimental group and used either docetaxel or a similar regimen of chemotherapy in the control group. During the literature search, we excluded trials that did not fit our clinical question (mainly excluding oncogene-addicted NSCLC patients or patients receiving upfront therapy); no further selection based on histology, performance status, or other clinical characteristics was applied. Owing to the method of our analysis aimed at reconstructing individual patient data, another inclusion criterion was the availability of a Kaplan–Meier graph, comparing the two above-mentioned treatment options based on the endpoint of OS. Details of the reasons for excluding some studies and the relative numbers can be found in the PRISMA flowchart in Figure 1.

### Reconstruction of Individual Patient Data from Kaplan–Meier Curves and Statistical Analysis

Our analysis, performed by application of the online version of the IPDfromKM method, included a first phase in which the graph of each Kaplan–Meier curve was digitized using Webplotdigitizer (version 4 online; https://automeris.io/, accessed on 10 August 2024), and a second phase in which the AI algorithm reconstructed individual patient data (separately for each curve evaluated in the analysis) from the x–y coordinates deriving from the digitized KM curves [13] (https://www.trialdesign.org/one-page-shell.html#IPDfromKM, version 1.2.3.0, accessed 10 August 2024). Once these databases of reconstructed patients had been created, indirect comparisons were made between the combination treatments and the standard of care, using the same statistical tests (e.g., Cox multiple regression model) as in studies based on “real” patients.

Design of the analysis. Regarding the design of our analysis, since five trials were selected as sources of clinical material, we first selected OS as the endpoint of the statistical analysis and then performed an indirect comparison between the five arms pooled together of the studies treated with combination treatment versus the five arms of the same studies treated with the standard of care (docetaxel or a similar regimen of chemotherapy). The hazard ratio (HR), with a 95% confidence interval (CI), was the parameter for testing the superiority of combination treatments versus standard of care. Finally, the five control arms of the included trials were subjected to an assessment of cross-trial heterogeneity, which was based on Wald’s test and the likelihood ratio test. In the second phase, for the four RCTs for which the PFS-KM curve was available, the same design was used to analyze PFS as a secondary endpoint. In the present investigation, we used three statistical packages (“survival”, “survminer”, “survRM2”, and “readxl”) of the R-platform (version 4.3.2). Our study was registered in the Research Registry database (with the registration code 10930 issued on 21 December 2024).

## 3. Results

The PRISMA algorithm was used to select the trials eligible for our analysis. After selecting only RCT according to the design of our analysis, six eligible were identified [17,18,19,20,21,22]; Figure 1 shows the PRISMA algorithm. For OS analysis, we excluded the trials by Schoenfeld et al. [22], owing to the absence of a control arm with standard chemotherapy; as a result, five RCTs were included in our analysis (Table 1).

**Figure 2:**
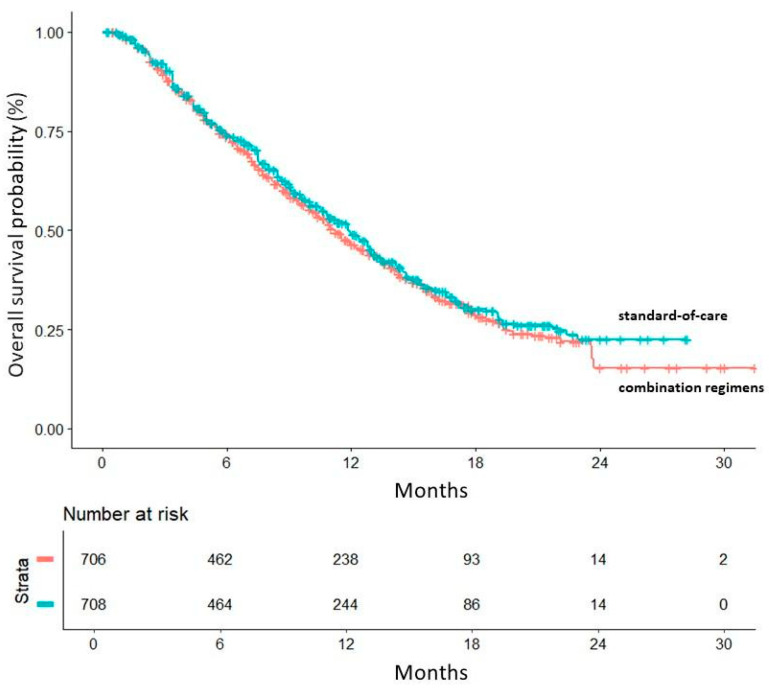
Main analysis: in red, combination regimens; in green, standard-of-care. Time in months.

The combination regimens evaluated in these trials included pembrolizumab + chemotherapy, pembrolizumab + ramucirumab, sitravatinib + nivolumab, atezolizumab + cabozantinib, and canakinumab + docetaxel. Figure 2 describes the results of our main analysis, which shows that the pooled curves of active and control arms are largely superimposed with one another. The HR for the comparison of combination treatments vs. standard of care was HR = 1.066 (95%CI, 0.9311 to 1.221; *p* = 0.35). Among the five control arms (Figure S1 in the Supplementary Material), the level of cross-trial heterogeneity was remarkably low (likelihood ratio test = 3.76 on 4 df; *p* = 0.40; Wald test = 3.83 on 4 df, *p* = 0.40).

**Figure 3:**
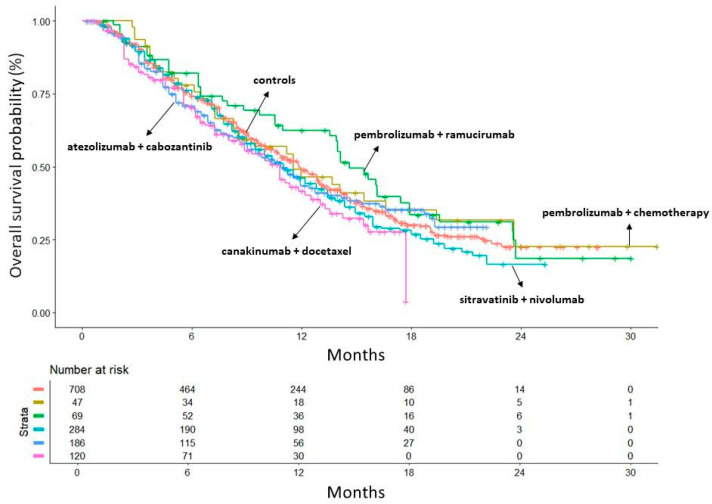
Overall survival: comparison of each of the 5 treatment arms with the 5 control arms pooled together. In red: the 5 control arms pooled together; in gold: trial by Jung et al. (pembrolizumab + chemotherapy) [17]; in green: trial by Reckamp et al. (ramucirumab + pembrolizumab) [18]; in light blue: trial by Borghaei et al. (sitravatinib + nivolumab) [19]; in dark blue: trial by Neal et al. (atezolizumab + cabozantinib) [20]; in purple: trial by Paz-Res et al. (canakinumab + docetaxel) [21]. Time in months.

**Figure 4:**
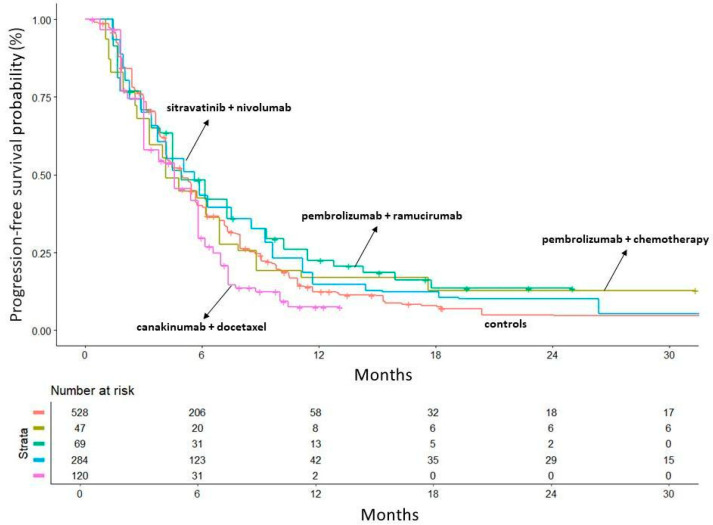
Progression-free survival: comparison of each of the 4 treatment arms with the 5 pooled control arms. In red: the 5 control arms pooled together; in gold: trial by Jung et al. (pembrolizumab + chemotherapy) [17]; in green: trial by Reckamp et al. (ramucirumab + pembrolizumab) [18]; in light blue: trial by Borghaei et al. (sitravatinib + nivolumab) [19]; in dark blue: trial by Neal et al. (atezolizumab + cabozantinib) [20]; in purple: trial by Paz-Res et al. (canakinumab + docetaxel) [21]. Time in months.

Figure 3 compares the OS pattern between each of the five treatment groups with the five control arms pooled together. Among the five treatment groups, the arm of the trial by Paz-Ares et al. [21] (canakinumab + docetaxel) showed the worse OS pattern (HR = 1.33, 95%CI 1.03–1.71) while the study by Reckamp et al. [18] investigating ramucirumab + pembrolizumab demonstrated the best OS (HR = 0.84, 95%CI 0.61 to 1.15); the other RCTs showed values of HR ranging from 0.91 to 1.11, but none of them reached the statistical nor the clinical significance compared to the standard of care. It should be noted that, unlike the other four trials, the trial by Borghaei et al. [19] included exclusively patients with non-squamous histology; however, both Figure 4 and Figure S1 show that this difference had no impact on the pattern of OS.

For the PFS analysis, we excluded the atezolizumab + cabozantinib RCT by Neal et al. because it does not provide a KM curve for PFS. In the subsequent analysis, both the combination of pembrolizumab + ramucirumab (HR = 0.81, 95%CI 0.6–1.07) and sitravatinib + nivolumab (HR = 0.89, 95%CI 0.77–1.04) appeared to provide a modest benefit compared to docetaxel, while canakinumab + docetaxel was confirmed as an ineffective combination (HR = 1.28, 95%CI 1.03–1.06) (Figure 4). Despite the modest benefit, none of the combinations reached statistical significance. The heterogeneity analysis performed on the control groups confirmed that the results from the different RCTs were comparable and that the heterogeneity was low (likelihood ratio test = 3.72 on 3 df, *p* = 0.3, Wald test = 3.85 on 3 df, *p* = 0.3) (Figure S2 in the Supplementary Material). From a clinical perspective, the two best combinations did not significantly prolong median PFS compared to docetaxel [pembrolizumab + ramucirumab: median PFS, 4.94 months (95%CI 4.47–7.29); sitravatinib + nivolumab: median PFS, 5.62 months (95%CI 4.17–5.86); docetaxel median PFS, 4.92 months (95%CI 4.36–5.46)].

## 4. Discussion

Since the introduction of docetaxel in second-line treatment for NSCLC, numerous efforts have been made to enhance its efficacy. Notably, combining docetaxel with anti-angiogenic drugs, such as the anti-VEGFR agent ramucirumab or the multi-target TKI nintedanib, has shown improved outcomes, albeit with limited impact on overall survival [23,24]. However, these combinations were investigated prior to the widespread use of ICIs in first-line treatment, leaving limited data on their efficacy in patients previously treated with ICIs.

The need for effective treatments for patients with advanced NSCLC progressing after ICIs and platinum-based chemotherapy remains a significant unmet need. Various mechanisms underlying primary or acquired resistance to ICIs in NSCLC have been proposed, providing a biological rationale for therapeutic strategies to overcome resistance, including a combination of ICIs with anti-angiogenic drugs, tumor microenvironment modulators, adhesion molecules, or novel ICIs targeting different pathways beyond CTLA-4 and PD-1/PD-L1, such as TIGIT [25].

In this context, different treatment options have already been investigated in phase 3 trials, reviewed in a recent network meta-analysis by Wang et al. [12]. Given the complexity of the treatment framework, our analysis was deliberately simplified to address a fundamental question: does a novel combination regimen provide a survival benefit compared to the current standard-of-care, docetaxel, or similar chemotherapy agents? Our analysis focused on randomized controlled trials (RCTs) specifically addressing this question, identifying five relevant RCTs testing five drug combinations for second-line or later treatment. After reconstruction of individual patient data, our pooled analysis based on 5 combination treatment arms and 5 control arms showed that the combination regimens provided no significant benefit in terms of OS and PFS. By contrast, in the network meta-analysis by Wang et al. [12], the antiangiogenic therapy combined with ICIs [7,12] improved OS compared to standard of care (HR = 0.69; 95%CI, 0.51 to 0.92). This difference can be explained by the fact that our analysis excluded studies of Ghiringhelli et al. and Lin Wu et al. [26,27], included in the meta-analysis by Wang et al. These studies, which explored the role of anti-angiogenic therapy, respectively, investigating the efficacy of atezolizumab + bevacizumab ± radiotherapy and anlotinib + docetaxel, were excluded from our analysis because they have been published as a conference abstract, not reporting KM curves. However, consistent with the findings of Wang et al. [12], we observed the most favorable HRs with the addition of an anti-angiogenic antibody, such as ramucirumab, or an anti-angiogenic and immunostimulatory multi-target.

In addition to the inclusion of preliminary results, the lack of significance seen in our analysis may be partly due to methodological reasons, which highlight a limitation of conventional meta-analysis. In fact, meta-analyses combine HRs into a single analysis that does not account for differences in follow-up time, typically assuming that event risks are constant over time—a premise that is often inaccurate, especially in datasets involving immune checkpoint inhibitors (ICIs). In contrast, the IPDfromKM method addresses this issue by incorporating follow-up time and taking into account response patterns that become apparent at later time points, such as those seen with pembrolizumab + ramucirumab.

However, the IPDfromKM method also has some limitations, such as the impossibility of performing a subgroup analysis if a KM curve of the subgroups of interest is not available. Another relevant limitation of the present analysis is its focus on OS and PFS, without accounting for treatment toxicity. In fact, the toxicity profiles of novel combinations and their impact on the quality of life are particularly critical in pretreated patients, where the primary goal of the treatment is palliation. Nevertheless, data from included studies suggest that the toxicity of the investigated combinations was expected and manageable, with no new safety signals identified. The network meta-analysis by Wang et al. [12] examined a larger number of outcomes, such as toxicity, but much of the efficacy data relies on indirect comparisons, which are associated with lower evidence levels.

Beyond the methodological issues, our analysis offers a number of clinical insights.

With the early use of chemo–immunotherapy combinations in the first-line setting, the characteristics of patients entering the second-line treatment may differ from those receiving platinum-based chemotherapy only. Indeed, in our analysis, the median OS of patients treated with docetaxel exceeded that reported in the original study by Shepherd et al. [28]. Converseley, real-world evidence showed that the performance of taxane monotherapy in ICI-pretreated patients was similar to that observed in patients treated only with upfront platinum-based chemotherapy [29,30]. These discrepancies underscore the need for new studies investigating the long-term effects of ICIs on the tumor environment and their impact on subsequent treatments.

In conclusion, our analysis did not identify any combination treatment with significantly superior efficacy to standard docetaxel for non-oncogene-addicted NSCLC with disease progression after ICIs and platinum-based chemotherapy. Treating these patients remains an urgent unmet need. However, our findings, together with Wang et al. [12], suggest that angiogenic agents may hold promise for future combinations with ICIs in this setting. Additionally, alternative strategies are emerging, aiming to transform a “cold” immune microenvironment into a “hot” one by enhancing anti-tumor lymphocyte infiltration. These approaches include novel drug classes such as bi-specific T-cell engagers (BiTEs) and antibody-drug conjugates (ADCs) [31,32]. Regardless of the future direction for second-line treatments in NSCLC patients pretreated with chemo–immunotherapy, robust analytical methods with clear, interpretable results remain valuable and important for effective decision-making. In this context, the IPDfromKM approach offers several advantages, including a multi-KM curve for immediate interpretation, while keeping the statistical analysis robust.

## Figures and Tables

**Table 1:** RCTs included in our analysis: main characteristics and OS data. Endpoint, death for any cause.

Study (Author, Year)	Study Type	Histology (Intervention Group vs. Controls)	Metastatic Sites (Intervention Group vs. Controls)	Intervention vs. Controls	Follow-Up Duration (Months)	HR for OS (95% CI) *	Total Number of Events/Patients (n/N) for OS	
							Treatment Group	Controls
1. Jung et al., 2022, NCT03656094, [17]	RCT	Adenocarcinoma: N = 26 (55.3%), vs. N = 25 (49.0%); squamous cell carcinoma: N = 20 (42.6%) vs. N = 25 (49.0%); pleomorphic carcinoma: N = 1 (2.2%) vs. N = 1 (2.0%)	Brain, N = 12 (25.5%) vs. N = 10 (19.6%); liver, N = 5 (10.6%) vs. N = 5 (9.8%); bone, N = 7 (14.9%) vs. N = 11 (21.6%)	Pembrolizumab + chemotherapy vs. chemotherapy ^$^	30	1.09 (0.66–1.83)	31/47	29/51
2. Reckamp et al., 2022, NCT03971474, [18]	RCT	Adenocarcinoma: N = 39 (58%) vs. 36 (52%); squamous cell carcinoma: N = 27 (40%) vs. 28 (41%); other: N = 1 (1.5%) vs. N = 5 (7%).	At least one organ: N = 69 (100%) vs. N = 67 (100%)	Pembrolizumab + ramucirumab vs. standard-of-care ^§^	30	0.69 (0.51–0.92)	45/69	51/67
3. Borghaei et al., 2024, NCT03906071, [19]	RCT	Non-squamous cell carcinoma	Brain, N = 58 (20.4%) vs. N = 62 (21.2%)	Sitravatinib + nivolumab vs. docetaxel	42	0.86 (0.70–1.05)	110/284	128/293
4. Neal et al., 2023, NCT04471428, [20]	RCT	Squamous cell carcinoma: N = 48 (25.8%) vs. N = 44 (24.4%); non squamous cell carcinoma: N = 138 (74.2%) vs. N = 136 (75.6%)	At least one organ: N = 186 (100%) vs. N = 180 (100%)	Atezolizumab + cabozantinib vs. docetaxel	22	0.88 (0.68–1.16)	114/186	106/180
5. Paz-Ares et al., 2024, NCT03626545, [21]	RCT	Adenocarcinoma, n = 75 (63%) vs. n = 75 (64%); large cell carcinoma, n = 1 (1%) vs. n = 0; squamous cell carcinoma, n = 42 (35%) vs. n = 39 (33%); other, n = 2 (2%) vs. n = 3 (3%)	Not reported	Canakinumab + docetaxel vs. docetaxel	18	1.06 (0.76–1.48)	73/120	68/117

* These values of HR for OS are those reported by the authors in their original articles. ^$^ Chemotherapy: gemcitabine, pemetrexed, docetaxel, or vinorelbine. ^§^ Standard of care: docetaxel/ramucirumab, docetaxel, gemcitabine, and pemetrexed.

## Data Availability

The files saved in the format of IPDfromKM software (software version, 1.2.3.0) are available from the corresponding author upon request.

## Supplementary Materials

The following supporting information can be downloaded at: https://www.mdpi.com/article/10.3390/curroncol32010046/s1, Figure S1: Heterogeneity analysis based on the 5 control arms: in red, rial by Jung et al. [17]; in dark green, trial by Reckamp et al. [18]; in light green,  trial by Borghaei et al. [19]; in blue, trial by Neal et al. [20]; in purple, trial by Paz-Res et al. [21]. Time in months. Figure S2: Heterogeneity analysis based on the 4 control arms of the RCT included for PFS analysis: in red, trial by Jung et al. [17]; in dark green, ttrial by Reckamp et al. [18]; in light blue,  trial by Borghaei et al. [19]; in purple, trial by Paz-Res et al. [21]. Time in months.

## Abbreviations

CI	confidence interval
PFS	progression-free survival
HR	hazard ratio
ICI	immune checkpoint inhibitor
KM	Kaplan–Meier
NSCLC	non-small cell lung cancer
OS	overall survival
RCT	randomized controlled trial
