# Supplementary material for: Treatment Options for Advanced Non-Small Cell Lung Cancer After Failure of Previous Immune Checkpoint Inhibitors and Chemotherapy: Meta-Analysis of Five Randomized Controlled Trials
Source: Curr Oncol. 2025 Jan 17;32(1):46. doi: 10.3390/curroncol32010046 (PMC11763427; doi:10.3390/curroncol32010046)
Supplement: Supplementary file 1 [file curroncol-32-00046-s001.zip › curroncol-3334586-supplementary.pdf]

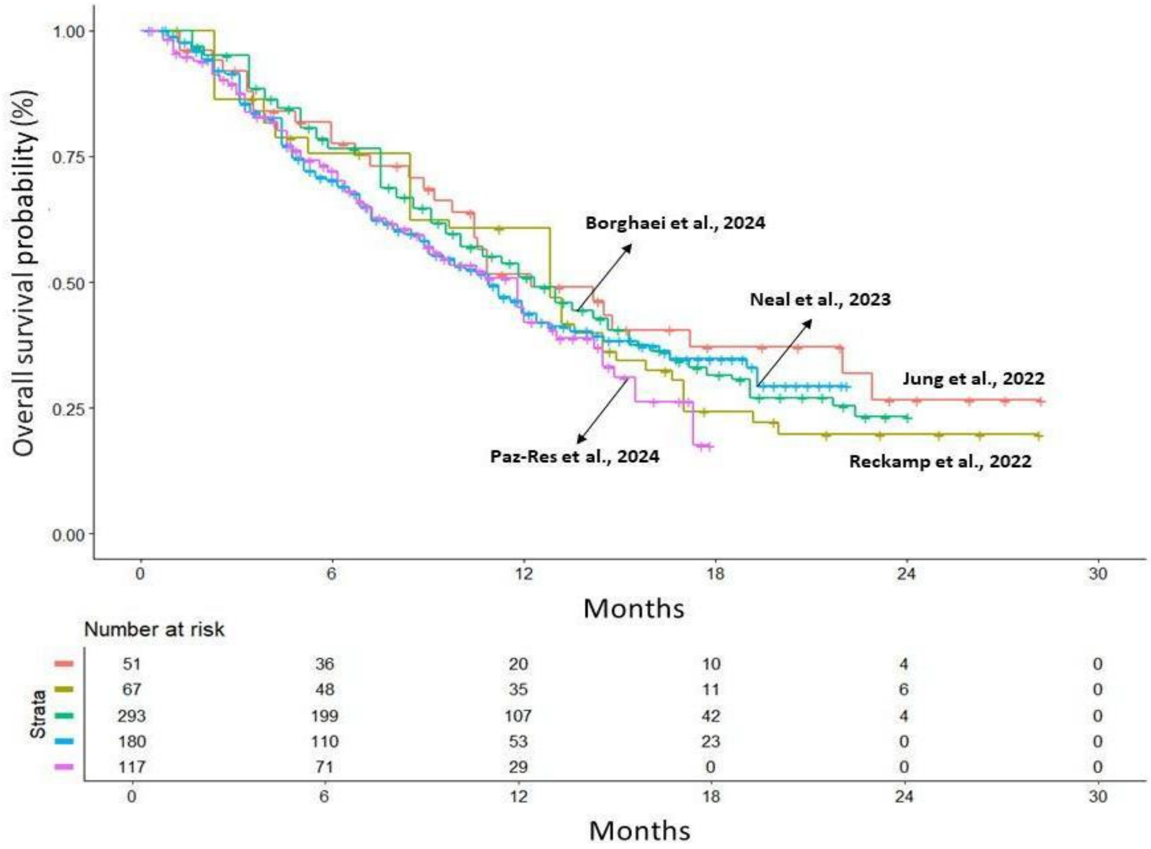

Figure S1: Heterogeneity analysis based on the 5 control arms: in red, rial by Jung *et al.* [17]; in dark green, trial by Reckamp *et al.* [18]; in light green, trial by Borghaei *et al.* [19]; in blue, trial by Neal *et al.* [20]; in purple, trial by Paz-Res *et al.* [21]. Time in months.

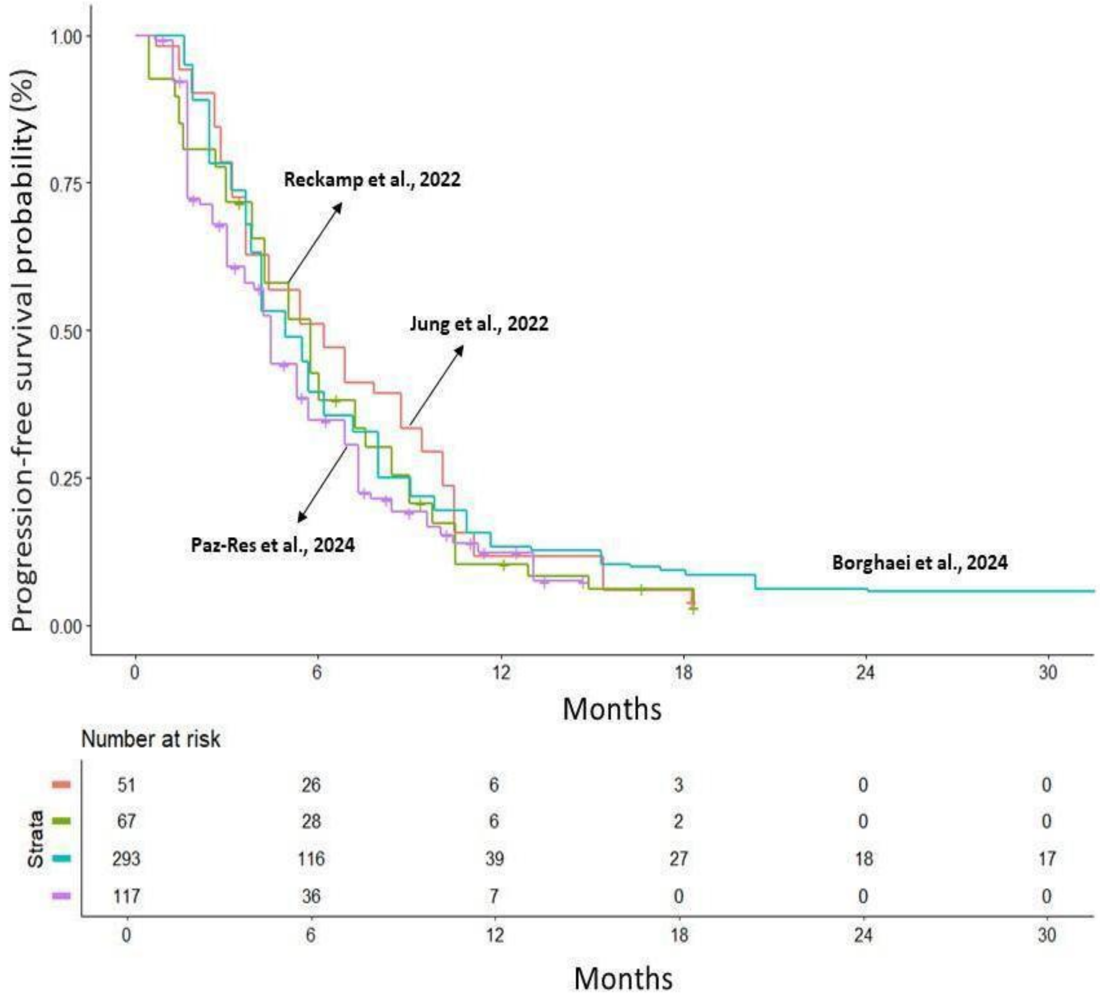

Figure S2: Heterogeneity analysis based on the 4 control arms of the RCT included for PFS analysis: in red, trial by Jung *et al.* [17]; in dark green, ttrial by Reckamp *et al.* [18]; in light blue, trial by Borghaei *et al.* [19]; in purple, trial by Paz-Res *et al.* [21]. Time in months.
